# Carbon Material-Reinforced Polymer Composites for Bipolar Plates in Polymer Electrolyte Membrane Fuel Cells

**DOI:** 10.3390/polym16050671

**Published:** 2024-03-01

**Authors:** Alejandro Gomez-Sanchez, Víctor A. Franco-Luján, Hilda M. Alfaro-López, Laura Hernández-Sánchez, Heriberto Cruz-Martínez, Dora I. Medina

**Affiliations:** 1 Tecnológico Nacional de México, Instituto Tecnológico del Valle de Etla, Abasolo S/N, Barrio del Agua Buena, Santiago Suchilquitongo, Oaxaca 68230, Mexico; alejandro.gs@itvalletla.edu.mx (A.G.-S.);; 2 Instituto Politécnico Nacional, Escuela Superior de Ingeniería Mecánica y Eléctrica, E.S.I.M.E.-Zacatenco, I.E., Edificio 2, U.P.A.L.M., Lindavista, Gustavo A. Madero, Ciudad de México 07738, Mexico; halfarol@ipn.mx; 3 Tecnologico de Monterrey, Institute of Advanced Materials for Sustainable Manufacturing, Monterrey 64849, Nuevo Leon, Mexico

**Keywords:** graphene, carbon nanotubes, carbon fibers, United States Department of Energy

## Abstract

Bipolar plates (BPs) are one of the most important components of polymer electrolyte membrane fuel cells (PEMFCs) because of their important role in gas and water management, electrical performance, and mechanical stability. Therefore, promising materials for use as BPs should meet several technical targets established by the United States Department of Energy (DOE). Thus far, in the literature, many materials have been reported for possible applications in BPs. Of these, polymer composites reinforced with carbon allotropes are one of the most prominent. Therefore, in this review article, we present the progress and critical analysis on the use of carbon material-reinforced polymer composites as BPs materials in PEMFCs. Based on this review, it is observed that numerous polymer composites reinforced with carbon allotropes have been produced in the literature, and most of the composites synthesized and characterized for their possible application in BPs meet the DOE requirements. However, these composites can still be improved before their use for BPs in PEMFCs.

## 1. Introduction

The modern society heavily relies on fossil fuel energy. However, this energy source is finite, and the byproducts of fossil fuels are associated with environmental problems such as climate change [1,2]. Therefore, we must move toward renewable energy sources to reduce the impact of anthropogenic activities associated with conventional energy conversion and production. In this direction, hydrogen can be crucial as a clean energy carrier with higher energy density compared with conventional fuels [3,4]. Although hydrogen is the most abundant element in the universe [5], it is not the primary energy source available on the earth. Therefore, various technologies have been developed for its production [6,7], storage [8,9], and use [10,11] in an efficient and safe manner. 

For hydrogen utilization, polymer electrolyte membrane fuel cells (PEMFCs) are gaining considerable importance because they allow for a highly efficient conversion of the chemical energy contained in hydrogen into electrical energy [11,12,13]. However, we must enhance the performance and reduce the cost of several of their components to achieve massive use of PEMFCs [11,12,13]. In particular, bipolar plates (BPs) are one of the components that have attracted attention because of their importance in the gas and water management, electrical performance, and mechanical stability of PEMFCs [14,15,16]. Therefore, promising materials for use as BPs should meet several technical targets established by the United States Department of Energy (DOE) [17].

Thus far, many materials have been investigated and used for the design of BPs. Graphite is the most widely used material for BPs because of its satisfactory corrosion resistance, high thermal and electrical conductivities, and stable chemical properties, among other properties [15,16]. However, it suffers several drawbacks such as limited mechanical properties (brittleness), high weight and volume, high manufacturing cost, and poor machinability [15,16,17,18,19]. Therefore, to address these drawbacks, carbon material-reinforced polymer composites have been widely studied as BPs because they offer several advantages such as a light weight, easy machinability, and satisfactory corrosion resistance [15,18,19,20,21,22]. Thus far, polymer matrices have been reinforced via various types of carbon allotropes such as graphite, graphene, multiwalled carbon nanotubes (MWCNTs), and carbon fibers [15,16,18,19,20,21,22]. Several of the proposed composites meet the DOE requirements. In the light of the importance that composites have gained, several review articles have analyzed the use of carbon material-reinforced composites as BPs in PEMFCs [15,16,18,19,20,21,22,23,24]. In 2017, the generalities of various types of carbon–polymer composites were revised [19]. Recently, various models for predicting the electrical conductivity of conductive polymer composites were analyzed [20]. In another review, various materials studied as promising candidates for BPs were reviewed, including polymer-based composites [21]. More recently, a comprehensive review of the current investigation on various materials used for developing polymer composites for BPs was conducted [22]. However, a detailed review focused on the properties of carbon-reinforced polymer composites as BPs materials still does not exist. Therefore, in this review, we present the progress on the use of carbon material-reinforced composites as BPs materials in PEMFCs.

## 2. Carbon-Reinforced Polymer Composites

### 2.1. Carbon-Reinforced Phenolic Resin Composites

Phenolic resin-based composites have a large market vis à vis their thermostructural applications because of their decent heat and flame resistance, satisfactory hardness, chemical resistance, and low cost [25,26]. Nevertheless, phenolics are nonconductive, can be brittle, and have low resistance to tensile strength [27,28]. Therefore, their properties should be substantially improved for application in BPs. In the first instance, phenolic resin-based composites were reinforced with a carbon allotrope [29,30,31,32,33,34,35,36,37,38,39,40,41,42,43,44], and the most studied composites of this type are based on phenolic resin and graphite [29,30,31,32,33,34,35,36,37,38,39,40,41,42]. Various compositions of resin and graphite have been studied (see Table 1). The electrical properties of the composites improved as the concentration of graphite-based materials increased in them [29,30,31,32,33,34,38,39,40,41,42], which is directly associated with the satisfactory electrical properties of graphite [45,46]. In contrast, the flexural strength decreased as the concentration of graphite materials increased in the composites [30,31,32,33,37,39,42]. This decrease in flexural strength might be associated with the unremarkable mechanical properties of graphite [45,47]. Nevertheless, some studies did not report a direct relationship between the flexural strength and concentration of graphite-based materials in the composites [29,41]. 

Phenolic resin-based composites have also been reinforced with other carbon allotropes, such as carbon fibers [29,34,43], carbon black [42,43,44], and MWCNTs [43]. However, the results obtained for these composites are still controversial (see Table 1). Some studies reported an increase in through-plane [29,44] and in-plane conductivities [34,42] upon increasing the concentration of these carbon allotropes. Nevertheless, other studies reported a reverse trend [43]. Regarding flexural strength, in most studies, optimum results were obtained at a specific composition [29,43,44]. However, other studies reported deterioration in this property when the carbon allotropes concentration increased in the composites [42,43]. Interestingly, there are some studies on the corrosion properties of phenolic resin composites reinforced with a carbon allotrope [32,37]. For instance, the corrosion resistance properties of phenolic resin (20 wt.%) and graphite (80 wt.%) composite were explored [32]. This composite presented good anodic (0.69 μA/cm^2^) and cathodic (1.05 μA/cm^2^) current densities because the values are similar to those required by the DOE (1 μA/cm^2^) [17,21]. In another study, the corrosion properties of phenolic resin-expanded graphite composites were measured by varying the composition of the phenolic resin and expanded graphite [37]. The phenolic resin-expanded graphite composites exhibited better corrosion resistance properties than expanded graphite BPs. Also, the corrosion resistance properties improved upon increasing the concentration of phenolic resin in the composites due to the good corrosion resistance of phenolic resin [37].

The design of phenolic resin composites reinforced with two [29,32,35,36,38,45,48] or three [29,38,49] carbon allotropes was proposed to enhance the mechanical and electrical properties of phenolic resin-based composites reinforced with one carbon allotrope. Unfortunately, several of the studies only analyzed three compositions, which makes it difficult to observe a trend between the composite compositions and their properties (see Table 2). Fortunately, a detailed study was conducted on the properties of phenolic resin-based composites reinforced with three carbon allotropes with different compositions of phenolic resin and exfoliated graphite [49]. Interestingly, the electrical and mechanical properties improved with the increase in exfoliated graphite concentration (see Table 2) [49]. Also, various studies have shown that phenolic resin-based composites reinforced with two or three carbon allotropes have better electrical and mechanical properties than phenolic resin-based composites with a carbon allotrope. For instance, the electrical and mechanical properties of phenolic resin–graphite–MWCNTs composites were higher than those of phenolic resin–graphite composites [32,35,36]. In another study, the electrical and mechanical properties of phenolic resin–graphite–expanded graphite composites were higher than those of phenolic resin–expanded graphite BPs [38]. However, more detailed studies on phenolic resin-based composites reinforced with two and three carbon allotropes are required.

**Figure 1:**
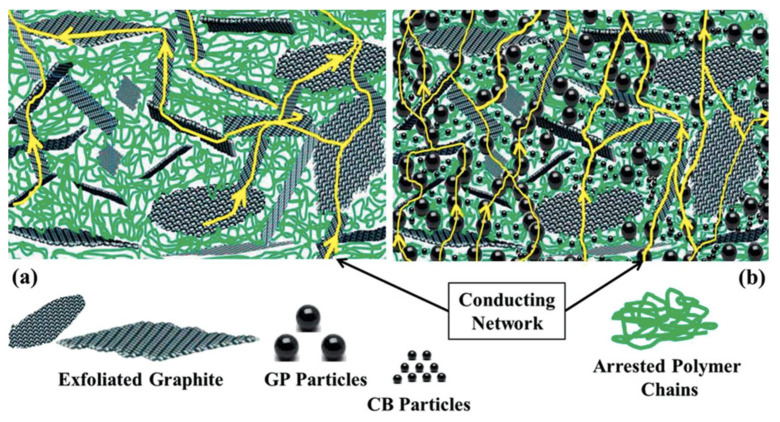
Schematic of electrical conduction mechanism in polymer composite containing (**a**) single and (**b**) multiple carbon fillers. Reproduced with permission from Reference [49].

Various phenolic resin composites reinforced with two or three carbon allotropes presented better electrical and mechanical properties than phenolic resin composites reinforced with a single carbon allotrope. These differences can be attributed to the distribution, composition, characteristics, and properties of the reinforcing materials [15,24]. For instance, the electrical properties of polymer composites depend on the conductive channels, the contact distance between the reinforcing materials, and the electrical conductivity of the reinforcing materials [49]. When the polymer composites are reinforced with various carbon allotropes (Figure 1b), the synergistic effect of the carbon allotropes produces a strong conducting network in the phenolic resin in comparison with a single carbon allotrope (Figure 1a).

### 2.2. Carbon-Reinforced Polypropylene Composites

Polypropylene has rapidly gained immense popularity in BPs because it is very cheap and flexible for molding and offers satisfactory mechanical properties with relatively decent resistance to impacts compared with other polymers [50,51,52]. However, it has a high electric resistance, oxidative degradation, and poor low-temperature impact strength [50,51,52]. Therefore, to be applied in BPs, it must be reinforced with carbon allotropes [53,54,55]. First, polypropylene was reinforced with a single carbon allotrope. Thus far, various studies have been conducted on polypropylene reinforced with a carbon allotrope [56,57,58,59,60,61,62,63,64,65,66], highlighting the use of graphite and MWCNTs. For example, polypropylene and graphite were produced in various ratios (32:68, 28:72, 24:76, 20:80, and 16:84 wt.%) [57]. Upon increasing the concentration of graphite in the composites, the through-plane conductivity tended to increase, whereas the flexural strength decreased [57]. In another study, various compositions of polypropylene–graphite (30:70, 25:75, 22.5:77.5, and 20:80 wt.%) were studied [62]. The in-plane conductivity tended to increase with increasing graphite concentration [62]. Additionally, various compositions of polypropylene–carbon black (98.5:1.5, 97:3, 95:5, 93:7, and 92:8 wt.%) were synthesized [59]. As in the case of polypropylene–graphite composites, upon increasing the concentration of carbon black in the composites, the through-plane conductivity increased, whereas the flexural strength tended to decrease [59]. For polypropylene–MWCNTs composites, the in-plane conductivity and flexural strength tended to increase in general upon increasing the concentration of MWCNTs [62,65,66]. On the corrosion properties of polypropylene composites reinforced with a carbon allotrope, [65], Ramírez-Herrera and collaborators studied the corrosion properties of polypropylene–MWCNTs composites by varying the composition of the polypropylene and MWCNTs [65]. The corrosion properties obtained for the polypropylene–MWCNTs composites were lower than those established by the DOE [65].

Polypropylene was reinforced with two or three carbon allotropes to further improve its electrical and mechanical properties [57,58,59,60,61,63,65,67,68,69,70,71]. In the first instance, polyprolylene was reinforced with two carbon allotropes [57,58,59,60,61,63,65,67,68,71], highlighting the use of graphite–carbon black and graphite–MWCNTs. Different studies were conducted on polyprolylene-based composites reinforced with graphite–carbon black [57,58,59,60,71]. For instance, polypropylene–graphite–carbon black composites were produced at various ratios (see Table 3) [57]. Upon increasing the concentration of graphite in the composites, the through-plane conductivity tended to increase, whereas the flexural strength decreased. In another study, polypropylene (20 wt.%) composites were reinforced with graphite–carbon black at various compositions (75:5, 70:10, 65:15, 60:20, 55:25, and 50:30 wt.%) [58]. The electrical properties (through-plane conductivity) of the composites tended to improve upon increasing the concentration of carbon black [58]. In general, a similar trend was observed in other studies [59,60], in which the through-plane conductivity tended to increase with an increase in the composition of carbon black in the composites. However, the mechanical properties did not exhibit any trend upon varying the composition of the composites [59]. Interestingly, the conductivity of polypropylene–graphite–carbon black was higher than that measured for polypropylene–graphite, which shows that the incorporation of carbon black is a satisfactory strategy to enhance the properties of these composites [58,59,60]. Some studies showed promising results for polyprolylene-based composites reinforced with graphite–MWCNTs [57,63,67]. For example, polypropylene–graphite composites at various ratios were reinforced using MWCNTs at 2 wt.% (see Table 3) [57]. Upon increasing the concentration of graphite in the composites, the through-plane conductivity tended to increase, whereas the flexural strength tended to decrease [57]. In another study, the electrical and mechanical properties of polypropylene–graphite–MWCNTs composites at various proportions (19:80:1, 18:80:2, and 16:80:4 wt.%) were analyzed [63]. The electrical (in-plane conductivity) and mechanical (flexural strength) properties improved upon increasing the concentration of MWCNTs in the composites [63]. On the corrosion properties for these composites, the corrosion properties of polypropylene–carbon fiber–MWCNTs composites were investigated [65]. The corrosion properties obtained for these composites were lower than those established by the DOE [65]. 

Polypropylene composites with three carbon allotropes were proposed to further reduce the graphite content in the composites (see Table 3). Thus far, several such studies have been conducted [67,69,70]. For instance, polypropylene–graphite–carbon fiber–carbon black composites were fabricated and studied. The in-plane conductivity increased upon increasing the concentration of carbon allotropes in the composites [69]. Additionally, the polypropylene–carbon black (20:25) composites were studied by varying the composition of the graphite and MWCNTs (54:1, 53:2, 52:3, 51:4, 50:5, 49:6, 48:7, 47:8, 46:9, and 45:10 wt.%) in the composites [70]. The electrical and mechanical properties tended to improve when the concentration of MWCNTs tended to increase in the composites. However, when the concentration of MWCNTs exceeded 6 wt.%, the electrical properties tended to deteriorate, whereas the flexural strength exhibited an oscillatory behavior for MWCNTs concentrations greater than 5 wt.% [70]. In another study, polypropylene–graphite–carbon black–MWCNTs composites at different compositions of graphite and carbon black were studied [67]. In general, upon increasing the carbon black concentration, the through-plane conductivity of the composites increased, whereas the flexural strength decreased [67]. 

### 2.3. Carbon-Reinforced Polyphenylene Sulfide Composites

Polyphenylene sulfide (PPS) has excellent chemical resistance, low degradation at high temperatures, and high rigidity. It also shows remarkable fatigue endurance and creep resistance, which have attracted extensive attention to it regarding its use for BPs [72,73,74]. However, PPS has a low elongation to break and low conductivity [75,76]. Therefore, to be used for BPs, it must be reinforced with carbon allotropes. In the first instance, PPS was reinforced with a carbon allotrope (see Table 4) [77,78,79,80]. For instance, PPS–graphite composites were studied at different concentrations [77]. Upon increasing the concentration of graphite in the composites, the in-plane conductivity increased, whereas the flexural strength decreased. A similar trend was observed in the electrical properties of PPS–mesocarbon composites [80]. In another study, PPS–graphene composites were produced at different ratios [79]. Upon increasing the concentration of graphene in the composites, the in-plane conductivity increased, whereas the flexural strength exhibited an oscillatory behavior [79]. 

In a bid to further improve the electrical and mechanical properties of PPS-based composites, this polymer has been reinforced with two carbon allotropes with promising results [78,79,80]. For example, PPS was reinforced at different ratios of graphite–carbon black. The through-plane conductivity tended to increase upon increasing the concentration of carbon black in the composites, which shows the importance of carbon black in the composites [78]. In addition, the PPS polymer was reinforced with different compositions of carbon black–graphene [79]. The flexural strength increased as the composition of graphene increased in the composites. The in-plane conductivities obtained for these composites are considerably different from the targets established by the DOE. On the corrosion properties for these composites, the corrosion properties of PPS–graphite–carbon black composites were investigated by varying the composition of the graphite and carbon black [78]. The corrosion properties obtained for the PPS–graphite–carbon black composites were similar to those required by the DOE [78]. Various PPS-based composites reinforced with one or two carbon allotropes meet the electrical and mechanical properties required by the DOE. However, the studies developed to date are still scarce. 

### 2.4. Carbon-Reinforced Polybenzoxazine Composites

The polybenzoxazine polymer has good thermal properties. However, this material exhibits high brittleness, which makes it difficult to use them to prepare films or complex structures [81,82,83]. Carbon allotrope-reinforced polybenzoxazine composites were proposed to improve the mechanical properties and processibility of these composites [84,85,86]. Thus far, some studies have been conducted on the use of polybenzoxazine composites reinforced with different types of carbon allotropes as materials for BPs (see Table 5) [87,88,89,90]. For instance, polybenzoxazine–graphite composites at different compositions of graphite were studied. With the increasing concentration of graphite in the composites, the in-plane conductivity increased, whereas the flexural strength decreased [87,88]. A similar trend was observed for polybenzoxazine–graphene composites [90]. Interestingly, the mechanical and electrical properties obtained for most of these composites were higher than those required by the DOE [87,88,89,90].

To reduce the graphite content in polybenzoxazine–graphene composites, as for other polymers, the strategy of incorporating other carbon allotropes in the composites has been established. Some studies were conducted on polybenzoxazine–graphene composites reinforced with two or three carbon allotropes. For instance, polybenzoxazine–graphite composites were reinforced with different concentrations of graphene [89]. The in-plane conductivity increased upon increasing the concentration of graphene in the composites. However, a higher flexural strength was observed in the polybenzoxazine 17%–graphite 80.5%–graphene 2.5% composite [89]. In another study, polybenzoxazine–graphite–graphene composites were reinforced with different concentrations of MWCNTs [91]. The in-plane conductivity increased as the concentration of MWCNTs increased in the composites. In addition, the electrical properties obtained for polybenzoxazine–graphite–graphene–MWCNTs were higher than those for polybenzoxazine–graphite–graphene ones [91]. These studies demonstrate the importance of incorporating more carbon allotropes into polybenzoxazine–graphite composites [89,91]. Various in polybenzoxazine-based composites reinforced with one or two carbon allotropes meet the electrical and mechanical properties required by the DOE. However, the investigations developed to date are still scarce.

### 2.5. Carbon-Reinforced Epoxy Resin Composites

Epoxy resin is also considered a polymer matrix for BPs because of its remarkably high adhesion strength, satisfactory heat resistance, good chemical and mechanical stabilities, easy mass production, and cost effectiveness [92]. However, epoxy resin BPs must exhibit better mechanical, electrical, and corrosion resistance properties to be applied as BPs [93]. Therefore, epoxy is generally reinforced with carbon allotropes to enhance these properties [24]. Recently, various studies have been conducted on epoxy resin reinforced with carbon allotropes [56,92,94,95,96,97,98], highlighting the use of graphite. Various ratios of epoxy resin–graphite were employed, ranging from ~20% to 80% graphite. Interestingly, the effect of the composition of epoxy resin–graphite composites on their electrical and mechanical properties was explored in detail. For instance, epoxy resin–graphite composites with different ratios (60:40, 50:50, and 40:60 wt.%) were investigated [94]. The in-plane conductivity, and flexural strength tended to increase upon increasing the concentration of graphite in the composites. A similar trend was observed in another study, in which different ratios of epoxy resin and graphite (40:60, 30:70, and 20:80 wt.%) were explored [96]. Interestingly, various synthesized materials comply with the DOE requirements [92,94,97].

Composites of epoxy resin reinforced with two carbon allotropes were investigated in a bid to reduce the graphite content in graphite-reinforced epoxy resin composites [94,95,96,98,99,100,101], as presented in Table 6. The most studied are epoxy resin composites reinforced with graphite–carbon black [94,95,99,100,101]. For instance, epoxy resins (20%) reinforced with various compositions of graphite–carbon black (60:20, 55:25, and 50:30 wt.%) were produced. The optimum results were observed for the epoxy resin (20%) reinforced with graphite–carbon black (55:25 wt.%) [101], highlighting that the in-plane conductivity was higher than that required by the DOE. In addition, epoxy resins (20 wt.%) reinforced with various concentrations of graphite–MWCNTs (79:1, 77.5:2.5, 75:5, 72.5:7.5, and 70:10 wt.%) were explored [96]. The optimum results were obtained for the epoxy resin (20 wt.%) reinforced with graphite–MWCNTs (75:5 wt.%). Interestingly, the electrical properties obtained for the epoxy resin reinforced with graphite–MWCNTs were superior to those of the epoxy resin reinforced with only graphite [96]. A similar trend was observed in another study, in which the electrical and mechanical properties of epoxy resin reinforced with carbon fiber–MWCNTs were superior to those of epoxy resin reinforced with only carbon fiber [98].

## 3. Carbon-Reinforced Two-Polymer Composites

Thus far, numerous single-polymer composites reinforced with one to three carbon allotropes have been explored, which have delivered promising results. Interestingly, two-polymer composites reinforced with carbon allotropes have also been explored [56,58,71,97,102,103]. In the first instance, two-polymer composites were reinforced with a carbon allotrope [56,58,97,102]. For example, epoxy resin–polyethylene composites were reinforced with various proportions of graphite [56,97]. The electrical properties improved upon increasing the concentration of graphite in the composites [56,97]. The optimum mechanical properties were achieved for a specific composition of epoxy resin, polyethylene, and graphite (see Table 7).

The synthesis and characterization of two-polymer composites reinforced with two or three carbon allotropes was proposed to improve the mechanical and electrical properties of composites formed by a carbon allotrope (see Table 8) [71,103]. In the first instance, two-polymer composites reinforced with two carbon allotropes (e.g., epoxy resin–polypropylene–graphite–carbon black composites) were proposed with satisfactory properties [103]. For example, these composites were studied at various concentrations of polymers and carbon allotropes [103]. The electrical properties improved as the concentration of the carbon allotropes increased in the composites [103]. The optimum mechanical properties were obtained for composites formed with epoxy resin (30%), polypropylene (10%), graphite (57%), and carbon black 3% [103]. In addition, two-polymer composites reinforced with three carbon allotropes were studied [71]. However, studies on these materials are scarce.

## 4. Discussion

### 4.1. Synthesis Methods

**Figure 2:**
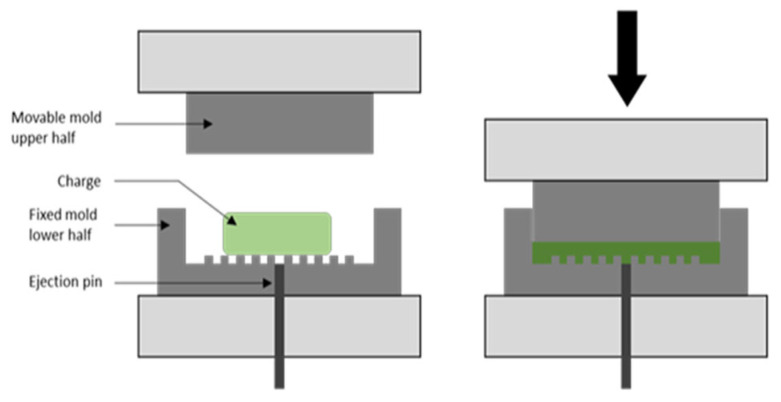
Compression molding technique to fabricate BPs. Reproduced with permission from Reference [104].

So far, several carbon allotrope-reinforced polymer composites have been produced (see Table 1, Table 2, Table 3, Table 4, Table 5, Table 6, Table 7 and Table 8), where several of these synthesized composites have the same or similar compositions. However, their mechanical and electrical properties differ substantially. These differences can be attributed to the synthesis conditions employed to produce these composites. Almost all analyzed polymer composites were produced using the compression molding technique (Figure 2). This method uses some parameters that have an influence on the characteristics and properties of the synthesized composites such as molding time, molding temperature, and molding pressure [104]. Therefore, it is important to consider these parameters for the production of polymer composites reinforced with carbon allotropes.

Molding time: It has been reported that this parameter substantially changes the characteristics and properties of polymer composites reinforced with carbon allotropes [30,87]. For instance, the electrical and mechanical properties of phenolic resin (15 wt.%) composites reinforced with graphite (85 wt.%) were investigated by varying the molding time (15, 30, 45, 60, 75, and 90 min) [30], and the best results were found with 60 min of molding time (142 S/cm and 61.6 MPa). In another study, polybenzoxazine (15 wt.%) composites reinforced with graphite (85 wt.%) were produced by varying the molding time (20, 30, 40, 60, 90, and 120 min) [87]. The maximum conductivity (228 S/cm) was measured at 60 min of molding time. While the maximum flexural strength (48 MPa) was obtained at 90 min [87]. 

Molding temperature: It has been documented that molding temperature substantially changes the electrical and mechanical properties of polymer composites reinforced with carbon allotropes [30,87,96]. For instance, the conductivity of a phenolic resin (15 wt.%) composite reinforced with graphite (85 wt.%) changed from 108 S/cm to 142 S/cm when the molding temperature increased from 220 to 240 °C, whereas the flexural strength increased from 53 MPa to 62 MPa when the temperature presented the same increase [30]. In another study, the conductivity of polybenzoxazine (15 wt.%) composite reinforced with graphite (85 wt.%) increased from 234 to 247 S/cm when the temperature changed from 160 °C to 200 °C. Also, the flexural strength increased from 34 to 44 MPa when the temperature increased from 160 °C to 200 °C [87]. 

Molding pressure: Some studies have demonstrated that the electrical and mechanical properties are directly related to the molding pressure [31,39,42,96,100]. For example, the conductivity and flexural strength of phenolic resin composites increased when the molding pressure increased [31,39,42]. The same trends were observed for epoxy resin composites [96,100].

### 4.2. Production Costs

Polymer composites reinforced with carbon allotropes are excellent candidates for use in BPs because their properties are superior to those required by the DOE. However, some of the carbon allotropes (e.g., MWCNTs and graphene) utilized to reinforce polymer matrices present challenges related to production costs. It is well documented in the literature that the production methods used to produce these carbon allotropes are still expensive because these structures were discovered recently [105,106,107]. For the year 2025, the DOE established cost targets of 2 USD/kW for BPs in PEMFCs [17,108]. Considering the current costs of graphene and MWCNTs, their real applications in BPs could be limited since BPs based on graphene-reinforced polymer materials are more expensive than graphite and metal BPs, and their costs could be much higher than those established by the DOE. Therefore, to ensure the use of composite materials reinforced with graphene and MWCNTs, it is necessary to have a method that allows for the production of these carbon structures in large quantities and with good quality, which could help to use these materials in BPs and, thus, comply with DOE’s cost targets.

### 4.3. Stability of BPs

The thermal stability of polymer composites reinforced with carbon allotropes is important for their use in BPs. However, it is well known that polymer-based composites can exhibit degradation problems at high temperatures. Therefore, it is essential to know the thermal stability of polymer composites reinforced with carbon allotropes at the PEMFCs operating temperatures (80–120 °C). Fortunately, there have been studies on the thermal stability of polymer composites reinforced with carbon allotropes at PEMFCs operating temperatures, and the results are promising [42,44,49,88]. For instance, the phenolic resin (45 wt.%) and graphite (55 wt.%) composite presented a 2.2 wt.% loss at 400 °C [42]. Also, the storage modulus was practically constant in a range from 30 to 100 °C [42]. In another study, the thermal stability of phenolic resin (varying the concentration) composites reinforced with exfoliated graphite (varying the concentration), carbon black (5 wt.%), and graphite (3 wt.%) was studied at 200 °C. The best results (0.03 wt.% loss) were obtained with the phenolic resin (57 wt.%)–exfoliated graphite (35 wt.%)–carbon black (5 wt.%)–graphite (3 wt.%) composite [49]. It has also observed that the storage modulus of the synthesized composites were similar when the temperature varied from 30 to 75 °C. Interestingly, it has also been shown that the incorporation of carbon allotropes improves the thermal stability of polymer composites [109,110,111,112,113,114,115]. According to the studies conducted on the thermal stability of polymer composites reinforced with carbon allotropes, these may not present serious degradation problems and may practically maintain the mechanical properties (storage modulus) at the operating temperatures of PEMFCs.

## 5. Conclusions and Future Directions

Carbon material–reinforced–polymer composites have been widely studied as BPs because they offer several advantages, such as a light weight, easy machinability, and a satisfactory corrosion resistance. From this detailed review, the following conclusions and future directions can be suggested:

(a) For single-polymer composites reinforced with carbon allotropes, phenolic resin, polypropylene, PPS, polybenzoxazine, and epoxy resin are the polymers more commonly used for BPs. However, more studies are required for PPS, polybenzoxazine, and epoxy resin-based composites since the studies developed to date show promising results.
(b) The single-polymer composites have been reinforced using various types of carbon allotropes, mainly graphite, carbon fibers, carbon black, carbon nanotubes, and graphene. However, it is necessary to extend the study on single-polymer composites reinforced with carbon nanotubes and graphene since these are popular in the literature for their extraordinary electrical and mechanical properties.
(c) Two-polymer composites with one, two, or three carbon allotropes have been partially explored with outstanding results. Therefore, more detailed studies on these composites should be conducted.
(d) Almost all composites were produced using the compression molding technique. Nevertheless, the use of additive manufacturing could be a good strategy to produce BPs using the composites analyzed in this review.
(e) Future studies should report on the properties required by the DOE and, thus, facilitate the analysis of the results.

## Figures and Tables

**Table 1:** Electrical and mechanical properties of phenolic resin reinforced with an allotrope of carbon.

Material (wt.%)	Through-Plane Conductivity (S/cm) > 20 [17]	In-Plane Conductivity (S/cm) > 100 [17]	Flexural Strength (MPa) > 25 [17]
Phenolic resin(90)-Graphite(10) [29]	0		71
Phenolic resin(80)-Graphite(20) [29]	2		70
Phenolic resin(70)-Graphite(30) [29]	3		98
Phenolic resin(60)-Graphite(40) [29]	15		97
Phenolic resin(50)-Graphite(50) [29]	77		82
Phenolic resin(40)-Graphite(60) [29]	90		80
Phenolic resin(30)-Graphite(70) [29]	105		70
Phenolic resin(20)-Graphite(80) [29]	110		68
Phenolic resin(35)-Graphite(65) [30]		9	
Phenolic resin(25)-Graphite(75) [30]		25	58
Phenolic resin(20)-Graphite(80) [30]		55	53
Phenolic resin(15)-Graphite(85) [30]		115	50
Phenolic resin(10)-Graphite(90) [30]		169	25
Phenolic resin(35)-Graphite(65) [31]		23	51
Phenolic resin(30)-Graphite(70) [31]		26	48
Phenolic resin(25)-Graphite(75) [31]		44	48
Phenolic resin (20)-Graphite(80) [31]		56	46
Phenolic resin (15)-Graphite(85) [31]		80	38
Phenolic resin(10)-Graphite(90) [31]		82	26
Phenolic resin(20)-Graphite(80) [32]	29	162	61
Phenolic resin(20)-Graphite(80) [33]	200		61
Phenolic resin(15)-Graphite(85) [33]	230		34
Phenolic resin(10)-Graphite(90) [33]	385		26
Phenolic resin(90)-Graphite(10) [34]		0	
Phenolic resin(80)-Graphite(20) [34]		2	
Phenolic resin(70)-Graphite(30) [34]		3	
Phenolic resin(60)-Graphite(40) [34]		15	
Phenolic resin(50)-Graphite(50) [34]		72	
Phenolic resin(40)-Graphite(60) [34]		95	
Phenolic resin(30-Graphite(70) [34]		105	
Phenolic resin(20)-Graphite(80) [34]		109	
Phenolic resin(20)-Graphite(80) [35]		175	51
Phenolic resin(35)-Graphite(65) [36]	10	80	40
Phenolic resin(90)-Expanded graphite(10) [29]	4		54
Phenolic resin(80)-Expanded graphite(20) [29]	65		59
Phenolic resin(70)-Expanded graphite(30) [29]	91		58
Phenolic resin(60)-Expanded graphite(40) [29]	105		65
Phenolic resin(50)-Expanded graphite(50) [29]	105		61
Phenolic resin(40)-Expanded graphite(60) [29]	107		46
Phenolic resin(30)-Expanded graphite(70) [29]	110		45
Phenolic resin(25)-Expanded graphite(75) [37]			65
Phenolic resin(20)-Expanded graphite(80) [37]			63
Phenolic resin(15)-Expanded graphite(85) [37]			62
Phenolic resin(60)-Expanded graphite(40) [38]		165	39
Phenolic resin(50)-Expanded graphite(50) [38]		225	43
Phenolic resin(40)-Expanded graphite(60) [38]		285	37
Phenolic resin(40)-Expanded graphite(60) [32]		80	132
Phenolic resin(35)-Expanded graphite(65) [32]		100	130
Phenolic resin(30)-Expanded graphite(70) [32]		130	122
Phenolic resin(25)-Expanded graphite(75) [32]		160	115
Phenolic resin(20)-Expanded graphite(80) [32]		180	109
Phenolic resin(15)-Expanded graphite(85) [32]		220	100
Phenolic resin(90)-Expanded graphite(10) [34]		2	
Phenolic resin(80)-Expanded graphite(20) [34]		18	
Phenolic resin(70)-Expanded graphite(30) [34]		65	
Phenolic resin(60)-Expanded graphite(40) [34]		95	
Phenolic resin(50)-Expanded graphite(50) [34]		103	
Phenolic resin(40)-Expanded graphite(60) [34]		104	
Phenolic resin(30)-Expanded graphite(70) [34]		106	
Phenolic resin(20)-Expanded graphite(80) [34]		112	
Phenolic resin(25)-Lump synthetic graphite(75) [39]		50	66
Phenolic resin(20)-Lump synthetic graphite(80) [39]		77	64
Phenolic resin(15)-Lump synthetic graphite(85) [39]		111	43
Phenolic resin(10)-Lump synthetic graphite(90) [39]		118	32
Phenolic resin(25)-Flake synthetic graphite(75) [39]		55	66
Phenolic resin(20)-Flake synthetic graphite(80) [39]		85	65
Phenolic resin(15)-Flake synthetic graphite(85) [39]		118	51
Phenolic resin(10)-Flake synthetic graphite(90) [39]		130	38
Phenolic resin(20)-Synthetic graphite(80) [32]		106	61
Phenolic resin(80)-Exfoliated graphite(20) [40]		3	
Phenolic resin(70)-Exfoliated graphite(30) [40]		32	
Phenolic resin(60)-Exfoliated graphite(40) [40]		123	
Phenolic resin(50)-Exfoliated graphite(50) [40]		168	
Phenolic resin(40)-Exfoliated graphite(60) [40]		227	
Phenolic resin(30)-Exfoliated graphite(70) [40]		308	
Phenolic resin(20)-Exfoliated graphite(80) [40]		500	
Phenolic resin(90)-Exfoliated graphite(10) [41]		10	45
Phenolic resin(80)-Exfoliated graphite(20) [41]		12	46
Phenolic resin(70)-Exfoliated graphite(30) [41]		125	46
Phenolic resin(60)-Exfoliated graphite(40) [41]		160	48
Phenolic resin(50)-Exfoliated graphite(50) [41]		310	54
Phenolic resin(40)-Exfoliated graphite(60) [41]		375	48
Phenolic resin(30)-Exfoliated graphite(70) [41]		460	46
Phenolic resin(20)-Exfoliated graphite(80) [41]		640	37
Phenolic resin(70)-Flake graphite(30) [42]		116	43
Phenolic resin(65)-Flake graphite(35) [42]		134	42
Phenolic resin(60)-Flake graphite(40) [42]		161	39
Phenolic resin(55)-Flake graphite(45) [42]		214	35
Phenolic resin(50)-Flake graphite(50) [42]		278	33
Phenolic resin(45)-Flake graphite(55) [42]		322	27
Phenolic resin(40)-Flake graphite(60) [42]		365	24
Phenolic resin(25)-Flake graphite(75) [39]		105	50
Phenolic resin(20)-Flake graphite(80) [39]		120	47
Phenolic resin(15)-Flake graphite(85) [39]		148	42
Phenolic resin(10)-Flake graphite(90) [39]		170	32
Phenolic resin(25)-Lump graphite(75) [39]		65	50
Phenolic resin(20)-Lump graphite(80) [39]		100	44
Phenolic resin(15)-Lump graphite(85) [39]		141	41
Phenolic resin(10)-Lump graphite(90) [39]		155	31
Phenolic resin(90)-Carbon fiber(10) [29]	17		77
Phenolic resin(80)-Carbon fiber(20) [29]	45		87
Phenolic resin(70)-Carbon fiber(30) [29]	60		150
Phenolic resin(60)-Carbon fiber(40) [29]	68		169
Phenolic resin(50)-Carbon fiber(50) [29]	71		175
Phenolic resin(40)-Carbon fiber(60) [29]	74		181
Phenolic resin(30)-Carbon fiber(70) [29]	80		90
Phenolic resin(20)-Carbon fiber(80) [29]	89		55
Phenolic resin(99)-Carbon fiber(1) [43]		260	53
Phenolic resin(97)-Carbon fiber(3) [43]		212	58
Phenolic resin(95)-Carbon fiber(5) [43]		204	60
Phenolic resin(93)-Carbon fiber(7) [43]		203	57
Phenolic resin(91)-Carbon fiber(9) [43]		198	56
Phenolic resin(90)-Carbon fiber(10) [34]		15	
Phenolic resin(80)-Carbon fiber(20) [34]		28	
Phenolic resin(70)-Carbon fiber(30) [34]		45	
Phenolic resin(60)-Carbon fiber(40) [34]		60	
Phenolic resin(50)-Carbon fiber(50) [34]		71	
Phenolic resin(40)-Carbon fiber(60) [34]		75	
Phenolic resin(30)-Carbon fiber(70) [34]		79	
Phenolic resin(20)-Carbon fiber(80) [34]		95	
Phenolic resin(95)-Carbon black(5) [44]	0		30
Phenolic resin(90)-Carbon black(10) [44]	0.02		37
Phenolic resin(85)-Carbon black(15) [44]	0.08		45
Phenolic resin(80)-Carbon black(20) [44]	0.15		50
Phenolic resin(75)-Carbon black(25) [44]	0.22		54
Phenolic resin(70)-Carbon black(30) [44]	0.31		51
Phenolic resin(65)-Carbon black(35) [44]	0.4		47
Phenolic resin(60)-Carbon black(40) [44]	0.45		43
Phenolic resin(97.5)-Carbon black(2.5) [43]		259	48
Phenolic resin(95)-Carbon black(5) [43]		309	46
Phenolic resin(92.5)-Carbon black(7.5) [43]		261	47
Phenolic resin(90)-Carbon black(10) [43]		208	24
Phenolic resin(98.5)-Carbon black(1.5) [42]		289	36
Phenolic resin(97)-Carbon black(3) [42]		320	33
Phenolic resin(95.5)-Carbon black(4.5) [42]		358	29
Phenolic resin(94)-Carbon black(6) [42]		354	26
Phenolic resin(92.5)-Carbon black(7.5) [42]		335	24
Phenolic resin(99)-MWCNTs(1) [43]		264	49
Phenolic resin(98)-MWCNTs(2) [43]		289	55
Phenolic resin(97)-MWCNTs(3) [43]		268	55
Phenolic resin(96)-MWCNTs(4) [43]		258	60
Phenolic resin(95)- MWCNTs(5) [43]		201	61

**Table 2:** Electrical and mechanical properties of phenolic resin reinforced with two and three carbon allotropes.

Material (wt.%)	Through-Plane Conductivity (S/cm) > 20 [17]	In-Plane Conductivity (S/cm) > 100 [17]	Flexural Strength (MPa) > 25 [17]
Phenolic resin(9.6)-Graphite(86.4)-Carbon fiber(4) [48]		242	36
Phenolic resin(9.4)-Graphite(84.6)-Carbon fiber(6) [48]		202	39
Phenolic resin(9.2)-Graphite(82.8)-Carbon fiber(8) [48]		230	37
Phenolic resin(9)-Graphite(81)-Carbon fiber(10) [45]		182	35
Phenolic resin(80)-Graphite(10)-Expanded graphite(10) [29]	26		58
Phenolic resin(60)-Graphite(20)-Expanded graphite(20) [29]	86		62
Phenolic resin(40)-Graphite(30)-Expanded graphite(30) [29]	109		27
Phenolic resin(60)-Graphite(20)-Expanded graphite(20) [38]		275	45
Phenolic resin(50)-Graphite(25)-Expanded graphite(25) [38]		350	49
Phenolic resin(40)-Graphite(30)-Expanded graphite(30) [38]		420	42
Phenolic resin(80)-Graphite(10)-Carbon fiber(10) [29]	54		105
Phenolic resin(60)-Graphite(20)-Carbon fiber(20) [29]	56		134
Phenolic resin(40)-Graphite(30)-Carbon fiber(30) [29]	89		115
Phenolic resin(80)-Expanded graphite(10)-Carbon fiber(10) [29]	40		69
Phenolic resin(60)-Expanded graphite(20)-Carbon fiber(20) [29]	100		99
Phenolic resin(40)-Expanded graphite(30)-Carbon fiber(30) [29]	96		74
Phenolic resin(19.9)-Expanded graphite(79.6)-MWCNTs(0.5) [32]	27	181	100
Phenolic resin(19.8)- Expanded graphite(79.2)-MWCNTs(1) [32]	33	182	100
Phenolic resin(19.7)- Expanded graphite(78.8)-MWCNTs(1.5) [32]	22	180	95
Phenolic resin(19.6)- Expanded graphite(78.4)-MWCNTs(2) [32]	23	181	91
Phenolic resin(20)-Graphite(79.5)-MWCNTs(0.5) [35]		180	56
Phenolic resin(20)-Graphite(79)-MWCNTs(1) [35]		195	57
Phenolic resin(20)-Graphite(78.5)-MWCNTs(1.5) [35]		190	55
Phenolic resin(20)-Graphite(78)-MWCNTs(2) [35]		185	54
Phenolic resin(34.8)-Graphite(64.7)-MWCNTs(0.5) [36]	25	165	54
Phenolic resin(35)- Graphite(64)-MWCNTs(1) [36]	29	180	56
Phenolic resin(34.5)-Graphite(64)-MWCNTs(1.5) [36]	30	165	50
Phenolic resin(34)- Graphite(64)-MWCNTs(2) [36]	30	145	46
Phenolic resin(40)-Graphite(45)-Carbon fiber(10)-Expanded graphite(5) [29]	102		65
Phenolic resin(82)-Exfoliated graphite(10)-Carbon black(5)-Graphite(3) [49]	5	20	49.5
Phenolic resin(77)-Exfoliated graphite(15)-Carbon black(5)-Graphite(3) [49]	18	57	51.5
Phenolic resin(72)-Exfoliated graphite(20)-Carbon black(5)-Graphite(3) [49]	24	124	56
Phenolic resin(67)-Exfoliated graphite(25)-Carbon black(5)-Graphite(3) [49]	48	220	58
Phenolic resin(62)-Exfoliated graphite(30)-Carbon black(5)-Graphite(3) [49]	74	310	60
Phenolic resin(57)-Exfoliated graphite(35)-Carbon black(5)-Graphite(3) [49]	97	375	62
Phenolic resin(60)-Expanded graphite(20)-Graphite(16)-Carbon black(4) [38]		160	38
Phenolic resin(50)-Expanded graphite(25)-Graphite(20)-Carbon Black(5) [38]		255	42
Phenolic resin(40)-Expanded graphite(30)-Graphite(24)-Carbon Black(6) [38]		400	39

**Table 3:** Electrical and mechanical properties of polypropylene reinforced with two and three allotropes of carbon.

Material (wt.%)	Through-Plane Conductivity (S/cm) > 20 [17]	In-Plane Conductivity (S/cm) > 100 [17]	Flexural Strength (MPa) > 25 [17]
Polypropylene(20)-Graphite(75)-Carbon fiber(5) [61]		263	40
Polypropylene(20)-Graphite(70)-Carbon fiber(10) [61]		105	33
Polypropylene(20)-Graphite(65)-Carbon fiber(15) [61]		93	28
Polypropylene(20)-Graphite(60)-Carbon fiber(20) [61]		78	30
Polypropylene(30)-Graphite(67.5)-Carbon black(2.5) [57]	3		36
Polypropylene(25)-Graphite(72.5)-Carbon black(2.5) [57]	9		37
Polypropylene(20)-Graphite(77.5)-Carbon black(2.5) [57]	21		28
Polypropylene(15)-Graphite(82.5)-Carbon black(2.5) [57]	27		30
Polypropylene(20)-Graphite(75)-Carbon black(5) [58]	17		
Polypropylene(20)-Graphite(70)-Carbon black(10) [58]	21		
Polypropylene(20)-Graphite(65)-Carbon black(15) [58]	25		
Polypropylene(20)-Graphite(60)-Carbon black(20) [58]	30		
Polypropylene(20)-Graphite(55)-Carbon black(25) [58]	37		
Polypropylene(20)-Graphite(50)-Carbon black(30) [58]	29		
Polypropylene(57)-Graphite(40)-Carbon black(3) [59]	0.04		39
Polypropylene(54)-Graphite(40)-Carbon black(6) [59]	0.45		39
Polypropylene(51)-Graphite(40)-Carbon black(9) [59]	1		40.5
Polypropylene(48)-Graphite(40)-Carbon black(12) [59]	2		34
Polypropylene(37)-Graphite(60)-Carbon black(3) [59]	0.8		40.5
Polypropylene(34)-Graphite(60)-Carbon black(6) [59]	2.5		37.5
Polypropylene(31)-Graphite(60)-Carbon black(9) [59]	20		37.5
Polypropylene(28)-Graphite(60)-Carbon black(12) [59]	75		35
Polypropylene(20)-Graphite(75)-Carbon black(5) [60]	18		
Polypropylene(20)-Graphite(70)-Carbon black(10) [60]	21		
Polypropylene(20)-Graphite(65)-Carbon black(15) [60]	61		
Polypropylene(20)-Graphite(60)-Carbon black(20) [60]	140		
Polypropylene(20)-Graphite(55)-Carbon black(25) [60]	223		
Polypropylene(20)-Graphite(50)-Carbon black(30) [60]	122		
Polypropylene(28)-Graphite(65)-Carbon black(7) [71]		11	
Polypropylene(30)-Graphite(68)-MWCNTs(2) [57]	6		26
Polypropylene(25)-Graphite(73)-MWCNTs(2) [57]	9		28
Polypropylene(20)-Graphite(78)-MWCNTs(2) [57]	21		26
Polypropylene(15)-Graphite(83)-MWCNTs(2) [57]	49		22
Polypropylene(20)-Graphite(75)-MWCNTs(5) [67]	15		15
Polypropylene(19)-Graphite(80)-MWCNTs(1) [63]		340	23
Polypropylene(18)-Graphite(80)-MWCNTs(2) [63]		400	24
Polypropylene(16)-Graphite(80)-MWCNTs(4) [63]		525	25
Polypropylene(30)-Carbon fiber(65)-Graphene(5) [68]	3.12	3.49	162
Polypropylene(25)-Carbon fiber(70)-Graphene(5) [68]	4.93	2.73	172
Polypropylene(30)-Carbon fiber(65)-MWCNTs(5) [68]	11.51	7.18	165
Polypropylene(25)-Carbon fiber(70)-MWCNTs(5) [68]	14.76	11.12	99
Polypropylene(80)-Carbon fiber(10)-MWCNTs(10) [65]			43.1
Polypropylene(70)-Carbon fiber(15)-MWCNTs(15) [65]		8.2	45.3
Polypropylene(55)-Graphite(15)-Carbon fiber(15)-Carbon black(15) [69]		2.5	
Polypropylene(50)-Graphite(16.66)-Carbon fiber(16.66)-Carbon black(16.66) [69]		3.5	
Polypropylene(45)-Graphite(18.33)-Carbon fiber(18.33)-Carbon black(18.33) [69]		6	
Polypropylene(40)-Graphite(20)-Carbon fiber(20)-Carbon black(20) [69]		9	
Polypropylene(35)-Graphite(21.66)-Carbon fiber(21.66)-Carbon black(21.66) [69]		20	
Polypropylene(20)-Graphite(65)-Carbon fiber(10)-MWCNTs(5) [67]	12		20
Polypropylene(20)-Graphite(55)-Carbon fiber(20)-MWCNTs(5) [67]	12		15
Polypropylene(20)-Graphite(45)-Carbon fiber(30)-MWCNTs(5) [67]	11		14
Polypropylene(20)-Graphite(54)-Carbon black(25)-MWCNTs(1) [70]	114		16
Polypropylene(20)-Graphite(53)-Carbon black(25)-MWCNTs(2) [70]	140		17
Polypropylene(20)-Graphite(52)-Carbon black(25)-MWCNTs(3) [70]	145		23
Polypropylene(20)-Graphite(51)-Carbon black(25)-MWCNTs(4) [70]	146		27
Polypropylene(20)-Graphite(50)-Carbon black(25)-MWCNTs(5) [70]	150		30
Polypropylene(20)-Graphite(49)-Carbon black(25)-MWCNTs(6) [70]	160		27
Polypropylene(20)-Graphite(48)-Carbon black(25)-MWCNTs(7) [70]	130		23
Polypropylene(20)-Graphite(47)-Carbon black(25)-MWCNTs(8) [70]	110		25
Polypropylene(20)-Graphite(46)-Carbon black(25)-MWCNTs(9) [70]	109		26
Polypropylene(20)-Graphite(45)-Carbon black(25)-MWCNTs(10) [70]	105		28
Polypropylene(20)-Graphite(70)-Carbon black(5)-MWCNTs(5) [67]	7.5		44
Polypropylene(20)-Graphite(65)-Carbon black(10)-MWCNTs(5) [67]	13.5		20
Polypropylene(20)-Graphite(60)-Carbon black(15)-MWCNTs(5) [67]	15		17
Polypropylene(20)-Graphite(55)-Carbon black(20)-MWCNTs(5) [67]	14		10
Polypropylene(20)-Graphite(50)-Carbon black(25)-MWCNTs(5) [67]	13.5		9
Polypropylene(20)-Graphite(65)-Expanded graphite(10)-MWCNTs(5) [67]	15.5		20
Polypropylene(20)-Graphite(55)-Expanded graphite(20)-MWCNT(5) [67]	16.5		20

**Table 4:** Electrical and mechanical properties of polyphenylene sulfide reinforced with an allotrope of carbon.

Material (wt.%)	Through-Plane Conductivity (S/cm) > 20 [17]	In-Plane Conductivity (S/cm) > 100 [17]	Flexural Strength (MPa) > 25 [17]
Polyphenylene sulfide(50)-Flake graphite(50) [77]		2	75
Polyphenylene sulfide(40)-Flake graphite(60) [77]		39	72
Polyphenylene sulfide(35)-Flake graphite(65) [77]		60	68
Polyphenylene sulfide(30)-Flake graphite(70) [77]		82	66
Polyphenylene sulfide(25)-Flake graphite(75) [77]		108	48
Polyphenylene sulfide(20)-Flake graphite(80) [77]		120	45
Polyphenylene sulfide(10)-Flake graphite(90) [77]		130	35
Polyphenylene sulfide(15)-Graphite(85) [78]	36		
Polyphenylene sulfide(97.5)-Carbon fiber(2.5) [77]		123	55
Polyphenylene sulfide(95)-Carbon fiber(5) [77]		126	61
Polyphenylene sulfide(90)-Exfoliated graphene(10) [79]		0.03	77
Polyphenylene sulfide(80)-Exfoliated graphene(20) [79]		0.19	65
Polyphenylene sulfide(70)-Exfoliated graphene(30) [79]		0.56	75
Polyphenylene sulfide(60)-Exfoliated graphene(40) [79]		1.25	68
Polyphenylene sulfide(50)-Exfoliated graphene(50) [79]		1.58	70
Polyphenylene sulfide(40)-Exfoliated graphene(60) [79]		5.62	62
Polyphenylene sulfide(30)-Mesocarbon(70) [80]	9.31	64	45
Polyphenylene sulfide(25)-Mesocarbon(75) [80]	13.63	75	41
Polyphenylene sulfide(23)-Mesocarbon(77) [80]	15.77	80	40
Polyphenylene sulfide(20)-Mesocarbon(80) [80]	21.37	133.7	38
Polyphenylene sulfide(17)-Mesocarbon(83) [80]	22.52	141	32
Polyphenylene sulfide(15)-Mesocarbon(85) [80]	22.79	152	23

**Table 5:** Electrical and mechanical properties of polybenzoxazine reinforced with an allotrope of carbon.

Material (wt.%)	Through-Plane Conductivity (S/cm) > 20 [17]	In-Plane Conductivity (S/cm) > 100 [17]	Flexural Strength (MPa) > 25 [17]
Polybenzoxazine(20)-Graphite(80) [87]		198	54
Polybenzoxazine(15)-Graphite(85) [87]		203	50
Polybenzoxazine(10)-Graphite(90) [87]		206	34
Polybenzoxazine(5)-Graphite(95) [87]		210	15
Polybenzoxazine(60)-Graphite(40) [88]		0.2	85
Polybenzoxazine(50)-Graphite(50) [88]		3	75
Polybenzoxazine(40)-Graphite(60) [88]		12	62
Polybenzoxazine(30)-Graphite(70) [88]		106	59
Polybenzoxazine(25)-Graphite(75) [88]		215	55
Polybenzoxazine(20)-Graphite(80) [88]		250	50
Polybenzoxazine(17)-Graphite(83) [89]		284	58
Polybenzoxazine(90)-Graphene(10) [90]		2	66
Polybenzoxazine(80)-Graphene(20) [90]		3	60
Polybenzoxazine(70)-Graphene(30) [90]		10	55
Polybenzoxazine(60)-Graphene(40) [90]		39	54
Polybenzoxazine(50)-Graphene(50) [90]		130	52
Polybenzoxazine(40)-Graphene(60) [90]		360	42

**Table 6:** Electrical and mechanical properties of epoxy resin reinforced with two allotropes of carbon.

Material (wt.%)	Through-Plane Conductivity (S/cm) > 20 [17]	In-Plane Conductivity (S/cm) > 100 [17]	Flexural Strength (MPa) > 25 [17]
Epoxy resin(60)-Expanded graphite(30)-Carbon black(10) [99]	0.00276		
Epoxy resin(30)-Expanded graphite(60)-Carbon black(10) [99]	18.5		
Epoxy resin(40)-Expanded graphite(59)-Carbon black(1) [99]	37.4		
Epoxy resin(60)-Expanded graphite(35)-Carbon black(5) [94]		250	40
Epoxy resin(50)-Expanded graphite(45)-Carbon black(5) [94]	79	350	44
Epoxy resin(40)-Expanded graphite(55)-Carbon black(5) [94]		470	56
Epoxy resin(20)-Graphite(75)-Carbon black(5) [100]	1		48
Epoxy resin(20)-Graphite(70)-Carbon black(10) [100]	0.65		32.3
Epoxy resin(20)-Graphite(60)-Carbon black(20) [101]		80	7
Epoxy resin(20)-Graphite(55)-Carbon black(25) [101]		120	14
Epoxy resin(20)-Graphite(50)-Carbon black(30) [101]		55	4
Epoxy resin(40)-Expanded graphite(59.5)-Carbon black(0.5) [95]		37	
Epoxy resin(40)-Expanded graphite(59)-Carbon black(1) [95]		50	
Epoxy resin(40)-Expanded graphite(58)-Carbon back(2) [95]		42	
Epoxy resin(40)-Expanded graphite(57)-Carbon black(3) [95]		40	
Epoxy resin(60)-Expanded graphite(39.9)-Graphene(0.1) [99]	56		
Epoxy resin(60)-Expanded graphite(39.5)-Graphene(0.5) [99]	65.39		
Epoxy resin(20)-Graphite(79)-MWCNT(1) [96]	25	79	
Epoxy resin(20)-Graphite(77.5)-MWCNT(2.5) [96]	65	155	36
Epoxy resin(20)-Graphite(75)-MWCNT(5) [96]	75	180	45
Epoxy resin(20)-Graphite(72.5)-MWCNT(7.5) [96]	60	155	32
Epoxy resin(20)-Graphite(70)-MWCNT(10) [96]	50	130	26
Epoxy resin(97.5)-Carbon fiber(1.25)-MWCNT(1.25) [98]		120	46
Epoxy resin(97.75)-Carbon fiber(1.25)-MWCNT(1) [98]		95	44
Epoxy resin(98)-Carbon fiber(1.25)-MWCNT(0.75) [98]		62	47
Epoxy resin(98.25)-Carbon fiber(1.25)-MWCNT(0.5) [98]		59	34
Epoxy resin(98.5)-Carbon fiber(1.25)-MWCNT(0.25) [98]		52	36
Epoxy resin(40)-Expanded graphite(59.5)-Graphene(0.5) [95]		32	
Epoxy resin(40)-Expanded graphite(59)-Graphene(1) [95]		37	
Epoxy resin(40)-Expanded graphite(58)-Graphene(2) [95]		32.5	
Epoxy resin(40)-Expanded graphite(57)-Graphene(3) [95]		31	

**Table 7:** Electrical and mechanical properties of two polymers reinforced with an allotrope of carbon.

Material (wt.%)	Through-Plane Conductivity (S/cm) > 20 [17]	In-Plane Conductivity (S/cm) > 100 [17]	Flexural Strength (MPa) > 25 [17]
Epoxy resin(31.5)-Polypropylene(38.5)-Graphite(30) [56]	0.18	12.5	46
Epoxy resin(27)-Polypropylene(33)-Graphite(40) [56]	0.3	17	47
Epoxy resin(22.5)-Polypropylene(27.5)-Graphite(50) [56]	0.75	25	50
Epoxy resin(18)-Polypropylene(22)-Graphite(60) [56]	1.25	30	54
Epoxy resin(13.5)-Polypropylene(16.5)-Graphite(70) [56]	1.91	55	55
Epoxy resin(9)-Polypropylene(11)-Graphite(80) [56]	3.21	68	40
Epoxy resin(31.5)-Polyethylene(38.5)-Graphite(30) [97]	0.2	11	29
Epoxy resin(27)-Polyethylene(33)-Graphite(40) [97]	0.4	16	33
Epoxy resin(22.5)-Polyethylene(27.5)-Graphite(50) [97]	1.2	21	38
Epoxy resin(18)-Polyethylene(22)-Graphite(60) [97]	2.3	31	40
Epoxy resin(13.5)-Polyethylene(16.2)-Graphite (70) [97]	3	59	42
Epoxy resin(9)-Polyethylene(11)-Graphite(80) [97]	4.2	73	39
Epoxy resin(10)-Phenolic resin(85)-Graphite(5) [102]		137	26
Epoxy resin(15)-Phenolic resin(80)-Graphite(5) [102]		124	39
Epoxy resin(20)-Phenolic resin(75)-Graphite(5) [102]		102	46
Epoxy resin(25)-Phenolic resin(70)-Graphite(5) [102]		80	47
Epoxy resin(30)-Phenolic resin(65)-Graphite(5) [102]		54	47
Polypropylene(20)-Polyaniline(2)-Graphite(78) [58]	7.5		
Polypropylene(20)- Polyaniline(4)-Graphite(76) [58]	8		
Polypropylene(20)- Polyaniline(6)-Graphite(74) [58]	9.5		
Polypropylene(20)- Polyaniline(8)-Graphite(72) [58]	8		
Polypropylene(20)- Polyaniline(10)-Graphite(70) [58]	5		

**Table 8:** Electrical and mechanical properties of two polymers reinforced with two and three allotropes of carbon.

Material (wt.%)	Through-Plane Conductivity (S/cm) > 20 [17]	In-Plane Conductivity (S/cm) > 100 [17]	Flexural Strength (MPa) > 25 [17]
Epoxi resin(37.5)-Polypropylene(12.5)-Graphite(49)-Carbon black(1) [103]	0.5	50	45.5
Epoxi resin(33.75)-Polypropylene(11.25)-Graphite(53)-Carbon black(2) [103]	1	57	49
Epoxi resin(30)-Polypropylene(10)-Graphite(57)-Carbon black(3) [103]	2.5	65	52
Epoxi resin(26.25)-Polypropylene(8.75)-Graphite(61)-Carbon black(4) [103]	3	72	42
Epoxi resin(22.5)-Polypropylene(7.5)-Graphite(65)-Carbon black(5) [103]	4.6	75	33
Epoxi resin(18.75)-Polypropylene(6.25)-Graphite(69)-Carbon black(6) [103]	5.9	83	32
Epoxi resin (15)-Polypropylene(5)-Graphite(73)-Carbon black(7) [103]	8.4	90	29
Epoxi resin(11.25)-Polypropylene(3.75)-Graphite(77)-Carbon black(8) [103]	9	93	19
Polypropylene(23)-Polypropylene maleic anhydride(5)-Graphite(67)-Carbon black(5) [71]		5.3	44
Polypropylene(23)-Polypropylene maleic anhydride(5)-Graphite(66.5)-Carbon black(5.5) [71]		10	49
Polypropylene(23)-Polypropylene maleic anhydride(5)-Graphite(66)-Carbon black(6) [71]		15	51
Polypropylene(23)-Polypropylene maleic anhydride(5)-Graphite(65)-Carbon black(7) [71]		105	44
Polypropylene(18)-Polypropylene maleic anhydride(10)-Graphite(65)-Carbon black(7) [71]		28	39
Polypropylene(23)-Polypropylene maleic anhydride(5)-Graphite(66.5)-Carbon black(5)-Graphene(0.5) [71]		8	47
Polypropylene(23)-Polypropylene maleic anhydride(5)-Graphite(66)-Carbon black(5)-Graphene(1) [71]		10	52
Polypropylene(23)-Polypropylene maleic anhydride(5)-Graphite(65)-Carbon black(5)-Graphene(2) [71]		7	48

## Data Availability

Not applicable.
